# Editorial: Magnetic nanoparticles for biomedical imaging and therapy applications

**DOI:** 10.3389/fchem.2025.1670850

**Published:** 2025-08-14

**Authors:** Juan Gallo, Graeme J. Stasiuk, Anna Roig, Juan Pellico

**Affiliations:** ^1^ Advanced Magnetic Theranostic Nanostructures Lab, International Iberian Nanotechnology Laboratory, Braga, Portugal; ^2^ Department of Imaging Chemistry and Biology, School of Biomedical Engineering and Imaging Sciences, King’s College London, London, United Kingdom; ^3^ Institute of Materials Science of Barcelona (ICMAB-CSIC), Campus of the UAB, Barcelona, Spain

**Keywords:** magnetic nanopartcles, medical imaging, therapy, multifunctionality, clinical translation

Magnetic nanoparticles (MNPs) are highly relevant in nanomedicine due to their unique size-dependent magnetic properties and multifunctionality, enabling their use across diagnostic and therapeutic applications. Recent efforts have been directed toward optimizing their physicochemical properties, improving biocompatibility, and addressing critical biosafety issues to facilitate a successful clinical translation. This Research Topic brings together recent advances in the rational design, surface modification, and functional integration of MNPs, with particular emphasis on their uses in biomedical imaging and oncologic therapeutic strategies.

Liu et al. have presented an interesting study on the development of a multifunctional nanoplatform, MnO_2_/CDDP@PDA-Cy5.5, for the combined treatment and monitoring of thyroid cancer. The authors engineered hollow MnO_2_ nanoparticles loaded with cisplatin (CDDP) and coated them with polydopamine (PDA) and Cy5.5 to endow the system with stimuli-responsive drug release, biocompatibility, and optical imaging capabilities. The study demonstrates that under acidic and reductive tumor microenvironment (TME) conditions, the system effectively releases CDDP and generates Mn^2+^ for MRI imaging, enhancing both therapeutic and diagnostic outcomes while increasing the specificity of the treatment. The nanocarrier exhibited higher tumor accumulation and enhanced cytotoxicity against thyroid cancer cells compared to free CDDP, with minimal systemic toxicity. *In vivo* imaging and biodistribution data further corroborate an enhanced EPR-based tumor accumulation and favorable pharmacokinetics of the nanocarrier. The comprehensive characterization, including TEM, UV-vis, XPS, and MRI relaxivity studies, substantiates the robust design and function of the nanoplatform. This work contributes meaningfully to the field of nanotheranostics by integrating TME-responsive drug delivery with non-invasive image monitoring, presenting a compelling case for further translational research in solid tumors.

Malehmir et al. have contributed with a timely review that focuses on the hemocompatibility of magnetic nanoparticles (MNPs) and their diverse biomedical applications, including regenerative medicine, cancer therapy, drug delivery, and imaging. The novelty is grounded in the detailed integration of recent advances in green synthesis methods, highlighting environmentally friendly approaches to improving biocompatibility and reducing the toxicity of the MNP. This is a particularly distinct viewpoint from prior literature that often emphasizes physical or chemical synthesis without extensive attention to sustainable, non-toxic production routes. The review’s scientific merit lies in gathering reported knowledge on the interface between MNPs and blood components, an area critical for the clinical translation of nanoparticle-based therapies but frequently overlooked. By elaborating on the role of nanoparticle surface chemistry, especially surface charge, geometry, porosity, and polymer functionalization, in hemocompatibility, the authors provide insights into optimizing MNP design for safe systemic use. Moreover, the review consolidates essential findings regarding the hemolytic activity of various MNP formulations, emphasizing threshold levels considering the American Society for Testing and Materials standards. This can assist researchers developing blood-contacting nanomaterials. Integrating both biomedical potential and safety aspects, the review sets the stage for future studies, accelerating the clinical applicability of MNPs. Its value is heightened by bridging gaps between nanomaterial engineering, biological interactions, and clinical considerations.

Gao et al. have presented an approach to improving the anti-tumor efficacy of thymoquinone (TQ) against triple-negative breast cancer (TNBC) by encapsulating TQ in poly D,L-lactic-co-glycolic acid (PLGA) nanoparticles and further coating them with chitosan (CS). The innovation lies in the combined use of PLGA for sustained drug delivery and chitosan for enhanced cellular uptake, addressing the long-standing challenges of poor solubility and bioavailability of natural anticancer agents like thymoquinone. The manuscript contains an extensive characterization of the nanoparticles (particle size analysis, polydispersity, zeta potential, morphology, and encapsulation efficiency) to ensure the reproducibility of the nanomaterials features. Furthermore, it is demonstrated that CS-coated nanoparticles improve encapsulation efficiency and provide sustained, pH-sensitive drug release, which are critical characteristics for tumor targeting. Two TNBC cell lines (MDA-MB-231 and SUM-149) were used for the biological validation. The *in vitro* analysis served to explore the cellular uptake and controlled release profiles, showing a clear advantage of the CS-coated nanoparticles over free TQ and uncoated PLGA-TQ nanoparticles. Despite notable developments in cancer diagnosis and treatment, TNBC remains a major therapeutic challenge due to its aggressive nature, lack of estrogen and progesterone receptors, and lack of human epidermal growth factor receptor-2. TNBC is one of the most complex cancers to treat. Thus, this work addresses a clinically relevant problem and provides a foundation for further preclinical studies.

Finally, Davis et al. have reviewed the integration of organic functionalities into magnetic nanoparticles to create responsive materials for advanced biomedical and sensing applications. The authors focus on how the deliberate inclusion of organic moieties—such as ligands, polymers, and stimuli-responsive groups—can be used to tailor the properties of paramagnetic systems, particularly those incorporating gadolinium (Gd), manganese (Mn), or iron (Fe)-based nanomaterials. A central theme is the role of organic components in enhancing responsiveness to environmental cues such as pH, temperature, redox state, and enzyme presence, thereby enabling the dynamic modulation of magnetic resonance (MR) signals. The review highlights the synergy between inorganic paramagnetic cores and organic functional coatings or ligands. These organic components provide biocompatibility and colloidal stability and facilitate targeting, imaging contrast tuning, and therapeutic payload delivery. Through a series of representative examples, the authors demonstrate how the rational design of organic-functionalized nanostructures enables “smart” behavior—such as signal amplification or activation in specific physiological environments. This approach improves diagnostic accuracy and specificity in applications such as magnetic resonance imaging (MRI), theranostics, and biosensing. The review also outlines current challenges in the field, including synthetic complexity and *in vivo* stability, while identifying future directions aimed at translating these smart nanostructures into clinical platforms.

Overall, this Research Topic highlights the advantages of multifunctional magnetic nanoparticles for integrated cancer imaging and therapy, the versatility of hybrid systems incorporating organic components, and the increasing importance of sustainable green synthesis methods and particle hemocompatibility. Altogether, these represent key attributes of MNPs that hold strong promise for broader clinical application in the near future.

